# Bioinformatics analysis of prognostic value of *TRIM13* gene in breast cancer

**DOI:** 10.1042/BSR20190285

**Published:** 2019-03-22

**Authors:** Wei-xian Chen, Lin Cheng, Ling-yun Xu, Qi Qian, Yu-lan Zhu

**Affiliations:** 1 Department of Breast Surgery, The Affiliated Changzhou No.2 People’s Hospital of Nanjing Medical University, Changzhou 213000, Jiangsu Province, China; 2 Post-doctoral Working Station, The Affiliated Changzhou No.2 People’s Hospital of Nanjing Medical University, Changzhou 213000, Jiangsu Province, China

**Keywords:** Breast cancer, Biomarker, Prognosis, TRIM13

## Abstract

**Background:** Tripartite motif 13 (*TRIM13*) plays a significant role in various biological processes including cell growth, apoptosis, transcriptional regulation, and carcinogenesis. However, the prognostic significance of *TRIM13* gene in breast cancer treatment remains largely unclear. **Methods:** We performed a bioinformatics analysis of the clinical parameters and survival data as it relates to *TRIM13* in breast cancer patients using several online databases including Oncomine, bcGenExMiner, PrognoScan, and UCSC Xena. **Results:** We found that *TRIM13* was lower-expressed in different subtypes of breast cancer with respect to normal tissues. Estrogen receptor and progesterone receptor status were positively correlated with *TRIM13* level; whereas, the Scarff–Bloom–Richardson grade, Nottingham prognostic index, nodal status, basal-like status, and triple-negative status were negatively related to *TRIM13* expression in breast cancer patients with respect to normal individuals. Lower *TRIM13* expression correlated with worse distant metastasis free survival, relapse free survival, disease specific survival, and metastatic relapse free survival. We also confirmed a positive correlation between *TRIM13* and *RAB11FIP2* gene expression. **Conclusion:** Bioinformatics analysis revealed that *TRIM13* may be adopted as a promising predictive biomarker for prognosis of breast cancer. More in-depth experiments and clinical trials are needed to validate the value of *TRIM13* in breast cancer treatment.

## Introduction

Breast cancer remains the most common malignant tumor and a leading cause of cancer-related mortality in women worldwide [1]. Breast cancer treatment includes locoregional surgery, systemic chemotherapy, precision radiotherapy, endocrine therapy, biological targeting agents, and a combination of the above, mainly depending on clinical, pathological, and molecular features. Biomarkers are served as surrogates of these features for establishing prognostics and predicting outcomes. Finding more effective, sensitive, and specific biomarkers for the prognosis of patients is therefore urgently needed in breast cancer research [2].

The family of tripartite motif (TRIM) proteins (http://trimbase.tigem.it/) is one of the subfamilies of the RING type E3 ubiquitin ligases and contains more than 70 members in human chromosomes [3]. Accumulated evidence has suggested that some members of TRIM are involved in various biological processes including cell growth, apoptosis, transcriptional regulation, and carcinogenesis [4]. For example, knockdown of *TRIM66* inhibited malignant behavior and epithelial–mesenchymal transition in non-small-cell lung cancer, suggesting that *TRIM66* was an oncogene that promoted lung cancer progression [5]. *TRIM36* played a tumor suppressive role by reducing cell proliferation and migration as well as promoting apoptosis in prostate cancer [6]. Researchers and clinicians are increasingly regarding TRIM as important factor in cancer development [7,8].

*TRIM13*, a tumor suppressor gene in the 13q14 chromosome region, was frequently lost in various malignancies [9–13]. It has been reported to play a role in endoplasmic reticulum-associated degradation and to regulate autophagy caused by endoplasmic stress [14–16]. To date, caspase-8, Akt, L-type channels, and Nur77 have been shown to be substrates for *TRIM13*-mediated ubiquitination [16–19]. *TRIM13* over-expression caused stabilization of p53 and decrease of Akt kinase activity followed by induction of apoptosis [17]. Taken together, these findings suggest that *TRIM13* may not only function as a tumor suppressor, but also as a potential predictive biomarker for prognosis of cancer.

In the present study, we performed a bioinformatics analysis of the clinical parameters and survival data as it relates to *TRIM13* in breast cancer patients using several large online databases in order to evaluate the prognostic significance of *TRIM13* gene in breast cancer treatment.

## Materials and methods

### Oncomine

The Oncomine (http://www.oncomine.org) is a web-based data mining platform aimed at facilitating new discovery from genome-wide expression analysis [20]. *TRIM13* gene was queried in the database and the results were filtered by selecting breast cancer and Cancer versus Normal Analysis. The threshold included fold change ≥2 in expression between cancers and normal tissues, *P*-value ≤1E-4, and gene rank ≥ top 10%. The comparisons of mRNA levels of *TRIM13* in breast cancer and normal tissues in each individual dataset were analyzed using the Student’s *t* test. Gene co-expressed with *TRIM13* was also analyzed.

### bcGenExMiner

The Breast Cancer Gene-Expression Miner v4.1 (bcGenExMiner v4.1, http://bcgenex.centregauducheau.fr/BC-GEM), a mining tool of 36 published annotated genomics data (total of 5861 patients), was used to analyse *TRIM13* gene with clinical parameters such as age, Scarff–Bloom–Richardson (SBR) grade, Nottingham prognostic index (NPI), estrogen receptor (ER), progesterone receptor (PR), epidermal growth factor receptor-2 (HER-2), and nodal status using the expression module [21,22]. Relevance of *TRIM13* and metastatic relapse event was calculated using the prognostic module, and correlation of *TRIM13* and *RAB11FIP2* was assessed using the correlation module. Data were last updated on December 2017.

### PrognoScan

The PrognoScan (http://www.prognoscan.org/) is a popular online database for evaluating the biological relationship between gene expression and survival data including distant metastasis free survival, relapse free survival, and disease specific survival in breast cancer patients across a large collection of publicly available cancer microarray datasets [23]. This tool could automatically calculate *P*-value, HR, and 95% confidence intervals based on a certain gene expression. Data were last updated on March 2013.

### UCSC Xena

The UCSC Xena (http://xena.ucsc.edu/) is a functional genomics browser that provides visualization and integration for analyzing and viewing the public data hubs. The heat map was generated by data mining in The Cancer Genome Atlas (TCGA) Breast Cancer using the UCSC Xena browser.

## Results

### Reduced expression of *TRIM13* gene in breast cancer patients

**Figure 1:**
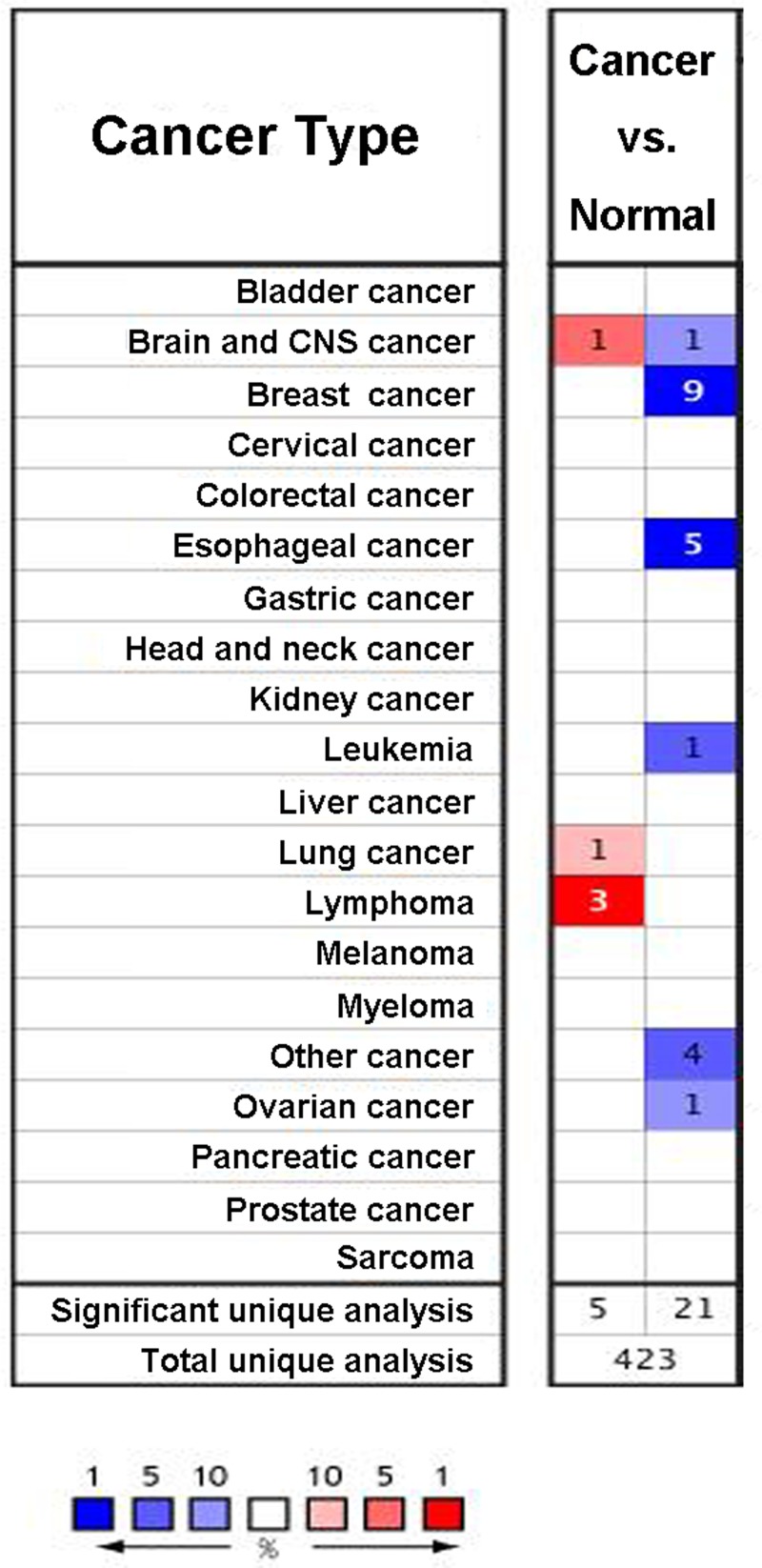
Expression of *TRIM13* gene in 20 common cancers versus paired normal tissues using the Oncomine database with the threshold of fold change ≥2, *P*-value ≤1E-4, and gene rank ≥ top 10% Red and blue respectively stand for the numbers of datasets with statistically significant (*P*<0.05) increased and decreased levels of *TRIM13* gene.

**Figure 2:**
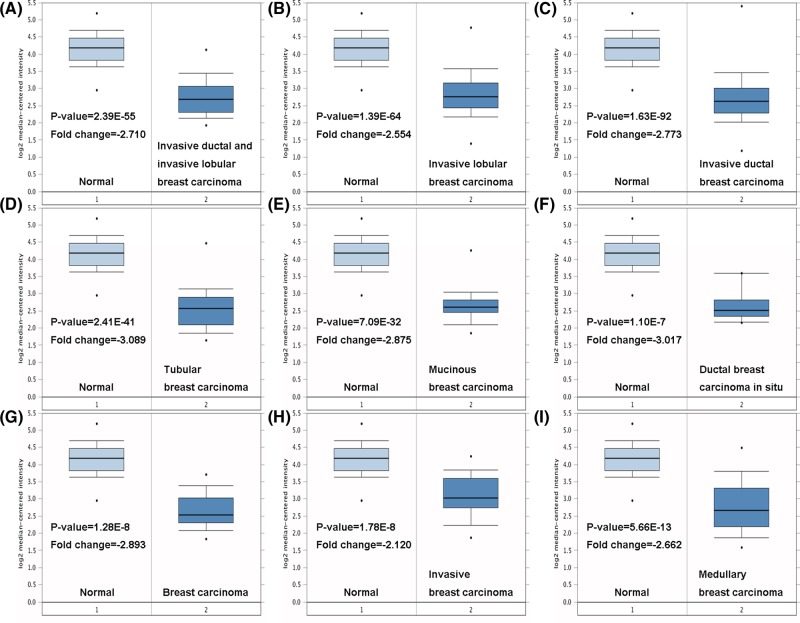
Box plot comparing *TRIM13* expression in normal individuals and breast cancer patients obtained from the Oncomine database Analysis is shown for invasive ductal and invasive lobular breast carcinoma (**A**), invasive lobular breast carcinoma (**B**), invasive ductal breast carcinoma (**C**), tubular breast carcinoma (**D**), mucinous carcinoma (**E**), ductal breast carcinoma *in situ* (**F**), breast carcinoma (**G**), invasive breast carcinoma (**H**), and medullary carcinoma (**I**).

We first measured the expression of *TRIM13* gene in 20 common types of cancer and compared its level to normal tissues using the Oncomine online database. Increased level of *TRIM13* (red) was found in brain cancer, lung cancer, and lymphoma; whereas, decreased level of *TRIM13* (blue) was observed in esophageal cancer, leukemia, ovarian cancer, and especially breast cancer (Figure 1). Oncomine analysis also revealed that *TRIM13* was significantly lower-expressed in invasive ductal and invasive lobular breast carcinoma, invasive lobular breast carcinoma, invasive ductal breast carcinoma, tubular breast carcinoma, mucinous carcinoma, ductal breast carcinoma *in situ*, breast carcinoma, invasive breast carcinoma, and medullary carcinoma with respect to normal individuals (Figure 2A–I, Table 1).

**Table 1:** *TRIM13* expression among different subtypes of breast cancer and normal individuals using the Oncomine database

Breast cancer subtype	*P*-value	*t* test	Fold change	Sample
Invasive ductal and invasive lobular breast carcinoma	2.39E-55	−23.164	−2.710	90
Invasive lobular breast carcinoma	1.39E-64	−22.594	−2.554	148
Invasive ductal breast carcinoma	1.63E-92	−37.680	−2.773	1556
Tubular breast carcinoma	2.41E-41	−21.724	−3.089	67
Mucinous breast carcinoma	7.09E-32	−20.140	−2.875	46
Ductal breast carcinoma *in situ*	1.10E-7	−11.852	−3.017	10
Breast carcinoma	1.28E-8	−10.636	−2.893	14
Invasive breast carcinoma	1.78E-8	−8.060	−2.120	21
Medullary breast carcinoma	5.66E-13	−10.639	−2.662	32

### *TRIM13* expression and clinical parameters of breast cancer patients

**Figure 3:**
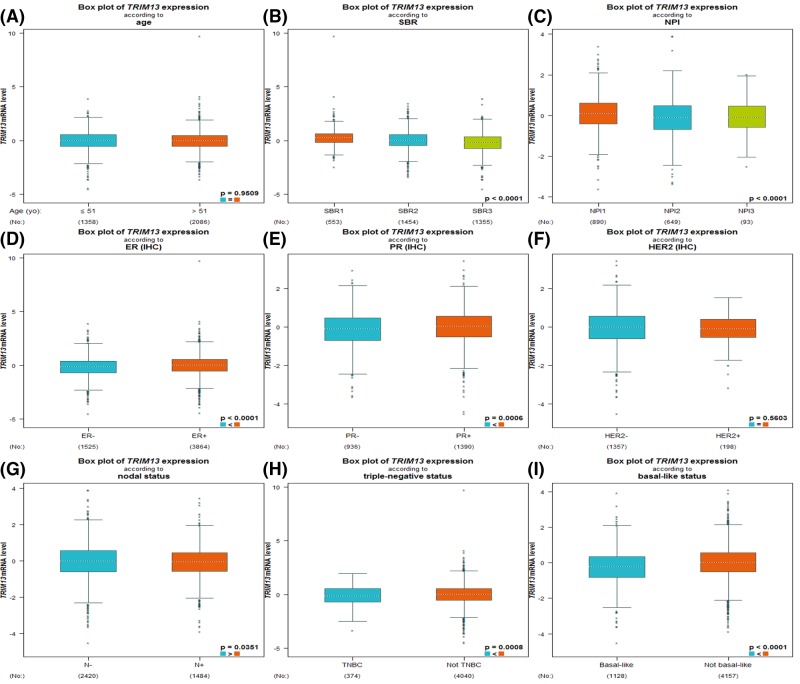
Box plot evaluating *TRIM13* expression among different groups of patients based on clinical parameters using the bc-GenExMiner software Analysis is shown for age (**A**), SBR grade (**B**), NPI index (**C**), ER (**D**), PR (**E**), HER-2 (**F**), nodal status (**G**), triple-negative status (**H**), and basal-like status (**I**).

We next evaluated *TRIM13* expression among different groups of patients based on several clinical parameters using the bc-GenExMiner software. For age criteria, no significant difference was found between ≤51 year and >51 year group (Figure 3A, Table 2). The SBR is a histological grade that evaluates tubule formation, nuclear characteristics of pleomorphism, and mitotic index. Based on tumor size, lymph node stage, and tumor grade, the NPI has been validated to stratify patients into additional prognostic groups. The SBR grade and NPI index are two accepted prognostic factors for breast cancer [24,25]; more advanced SBR grade and NPI index were associated with lower *TRIM13* level (Figure 3B,C). ER-positive or PR-positive breast cancer patients tended to express higher *TRIM13* gene compared with ER-negative or PR-negative patients (Figure 3D,E; Table 2). Regarding HER-2, there was no significant difference between positive and negative group (Figure 3F, Table 2). Patients with nodal-positive status showed reduced expression of *TRIM13* than nodal-negative patients (Figure 3G, Table 2). Moreover, *TRIM13* was significantly elevated in non-triple-negative and non-basal-like breast cancer patients with respect to triple-negative and basal-like breast cancer patients (Figure 3H,I; Table 2).

**Table 2:** Relationship between *TRIM13* expression and clinical parameters of breast cancer patients using the bc-GenExMiner database

Variables	No. of patients	*TRIM13* mRNA	*P*-value
**Age**			0.9509
≤51	1358	-	
>51	2086	-	
**ER**			<0.0001
Negative	1525	-	
Positive	3864	Increased	
**PR**			0.0006
Negative	936	-	
Positive	1390	Increased	
**HER-2**			0.5603
Negative	1357	-	
Positive	198	-	
**Nodal status**			0.0351
Negative	2420	Increased	
Positive	1484	-	
**Triple-negative status**			0.0008
Non-triple-negative	4040	Increased	
Triple-negative	374	-	
**Basal-like status**			<0.0001
Non-basal-like	4157	Increased	
Basal-like	1128	-	

### *TRIM13* expression and survival data of breast cancer patients

**Figure 4:**
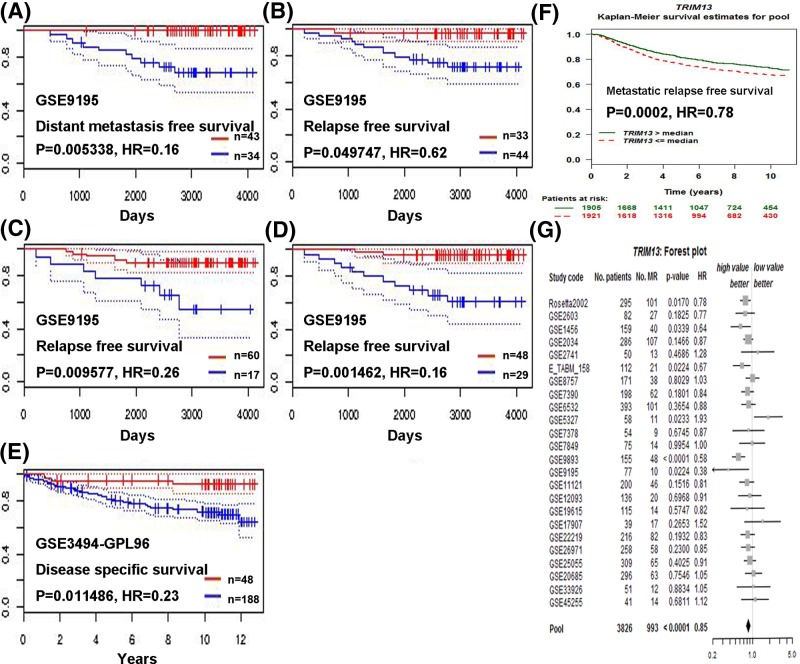
Survive curve and forest plot evaluating the prognostic value of *TRIM13* using the PrognoScan and bc-GenExMiner database Analysis is shown for distant metastasis free survival (**A**), relapse free survival for different probe (**B–D**), disease specific survival (**E**), metastatic relapse free survival (**F**), and forest plot (**G**). For A–E, red and blue stand for high and low expression of *TRIM13* gene, respectively.

We then investigated the prognostic value of *TRIM13* gene using the PrognoScan database. Higher expression of *TRIM13* (red) was significantly related to preferable distant metastasis free survival (Figure 4A, Table 3). Breast cancer patients with increased *TRIM13* level (red) presented better relapse free survival (Figure 4B–D, Table 3). Conversely, down-regulated *TRIM13* gene (blue) was strongly associated with worse disease specific survival (Figure 4E, Table 3). To further investigate the role of *TRIM13* in breast cancer prognosis, we used the bc-GenExMiner software to verify our findings. *TRIM13* was observed to be positively correlated with metastatic relapse free survival, as suggested by the survival curve and forest plot (Figure 4F–G, Table 3).

**Table 3:** *TRIM13* expression and survival data of breast cancer patients using the PrognoScan database

Dataset	Probe ID	End point	No.	Cox *P*-value	HR
GSE9195	203659_s_at	Distant metastasis free survival	77	0.005338	0.16 [0.04–0.58]
GSE9195	229943_at	Relapse free survival	77	0.049747	0.62 [0.38–1.00]
GSE9195	230192_at	Relapse free survival	77	0.009577	0.26 [0.09–0.72]
GSE9195	203659_s_at	Relapse free survival	77	0.001462	0.16 [0.05–0.49]
GSE3494–GPL96	203659_s_at	Disease specific survival	236	0.011486	0.23 [0.08–0.72]

### Co-expression of *TRIM13* gene

**Figure 5:**
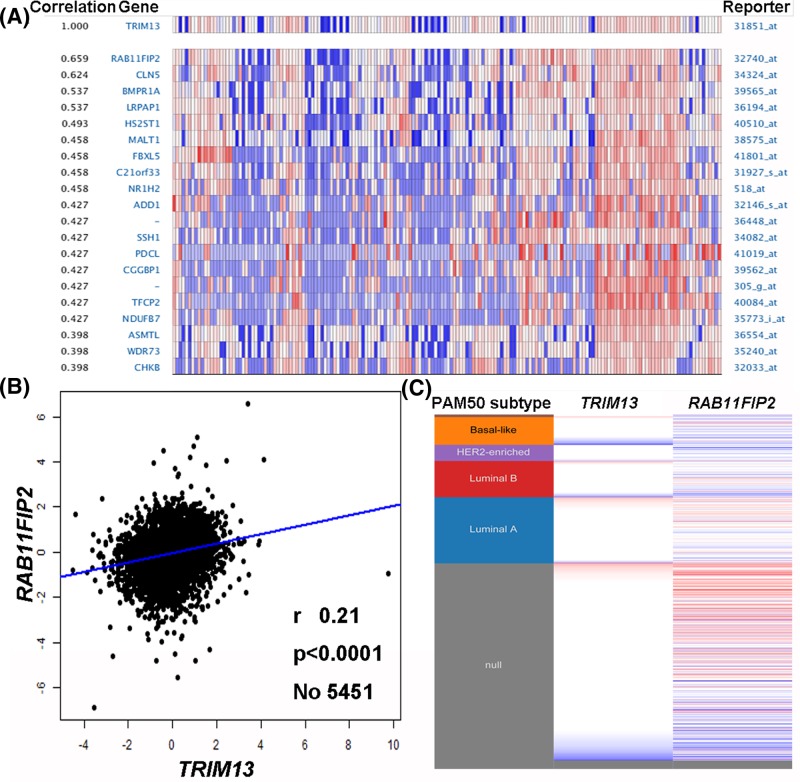
Co-expression of *TRIM13* gene (**A**) *TRIM13* co-expression of genes identified using the Oncomine database. (**B**) Correlation between *TRIM13* and *RAB11FIP2* expression in breast cancer analyzed using the bc-GenExMiner software. (**C**) Heat map of *TRIM13* and *RAB11FIP2* expression across a 50-gene qPCR assay (PAM50) breast cancer subtypes in the TCGA database generated using the UCSC Xena web-based tool.

We finally checked the co-expression of *TRIM13* gene using the Oncomine database. The co-expression profile of *TRIM13* was identified with a large cluster of 8603 genes across 174 cancer samples including 26 breast carcinomas (Figure 5A). Data mining using the bc-GenExMiner software revealed a positive correlation between *TRIM13* and *RAB11FIP2* expression (Figure 5B). After analyzing breast cancer patient data in the TCGA database using the UCSC Xena web-based tool, we also confirmed a positive correlation between *TRIM13* and *RAB11FIP2* expression, as shown in the heat map (Figure 5C).

## Discussion

*TRIM13*, a member of the TRIM family, has been reported to play a role in diverse cellular functions such as cell growth, immunity, and tumorigenesis including lipoma, leukemia, and myeloma [9–13,26,27]. *TRIM13* over-expression caused increased stability of p53 and decreased activity of Akt kinase, resulting in induction of apoptosis [17]. However, the significance of *TRIM13* expression in the development and prognosis of breast cancer remains largely unclear. To the best of our knowledge, this is the first study to identify *TRIM13* as a potential predictive biomarker for prognosis of breast cancer.

In the present work, we performed a bioinformatics analysis of the clinical parameters and survival data as it relates to *TRIM13* in breast cancer patients by pooling and analyzing several online tools. Oncomine database revealed that *TRIM13* was lower-expressed in invasive ductal and invasive lobular breast carcinoma, invasive lobular breast carcinoma, invasive ductal breast carcinoma, tubular breast carcinoma, mucinous carcinoma, ductal breast carcinoma *in situ*, breast carcinoma, invasive breast carcinoma, and medullary carcinoma with respect to normal tissues. For HER-2 criterion, there was no significant difference between positive and negative group. ER and PR status were positively correlated with *TRIM13* level. Conversely, SBR grade, NPI index, nodal status, basal-like status, and triple-negative status were negatively related to *TRIM13* expression in breast cancer patients in respect to normal individuals. Generally, breast cancer patients with ER or PR positive, nodal negative, non-basal-like or non-triple-negative status have a preferable outcome. Therefore, these results indicated that high expression of *TRIM13* may predict a better prognosis.

We further investigated the prognostic value of *TRIM13* in breast cancer using the PrognoScan and bc-GenExMiner software. The pooled results showed that lower *TRIM13* expression correlated with worse distant metastasis free survival, relapse free survival, disease specific survival, and metastatic relapse free survival. Such findings are in agreement with the notion of *TRIM13* as a tumor suppressor gene and a useful predictive biomarker for prognosis of breast cancer. We finally checked the co-expression of *TRIM13* gene using the Oncomine, bc-GenExMiner, and UCSC Xena web-based tools and found that *RAB11FIP2* was positively correlated with *TRIM13* expression. *RAB11FIP2*, a member of the RAB11 family of interacting proteins, exhibited potential tumor suppressor function since blocking of the *miR-192/215-RAB11FIP2* axis could inhibit gastric cancer progression [28]. This observation, along with our findings of *TRIM13* in survival information, provides evidence that *TRIM13* gene might inhibit tumor migration and invasion associated with *RAB11FIP2* expression.

In conclusion, the present work suggests that *TRIM13* was lower-expressed in different subtypes of breast cancer compared with normal tissues and was associated with several clinical parameters. *TRIM13* could be adopted as a promising predictive biomarker for prognosis of breast cancer with co-expressed *RAB11FIP2* gene. More in-depth experiments and clinical trials are needed to validate the value of *TRIM13* in breast cancer treatment.

## Abbreviations

ER: estrogen receptor
HER2: epidermal growth factor receptor-2
NPI: Nottingham prognostic index
PR: progesterone receptor
SBR: Scarff–Bloom–Richardson
TRIM: Tripartite motif 13
